# “Perceptions, expectations and satisfaction levels of occupational therapy students prior to and after practice placement and comparison of practice placement models”

**DOI:** 10.1186/s12909-019-1762-0

**Published:** 2019-08-29

**Authors:** Anat Golos, Esti Tekuzener

**Affiliations:** 1 Deputy Chair of School and Head of Undergraduate Studies, School of Occupational Therapy of Hadassah and the Hebrew University, Mt. Scopus, P.O.B: 24026, 91240 Jerusalem, Israel; 2 Clinical practitioner and Practice placement coordinator, School of Occupational Therapy of Hadassah and the Hebrew University, Mt. Scopus, P.O.B: 24026, 91240 Jerusalem, Israel

**Keywords:** Practice placement education, Professional development, Placement setting, Supervision, Community

## Abstract

**Background:**

Practice placements in occupational therapy are fundamental components in developing a student’s professional identity. Various models of placements are available to expose and expand students’ participation in various community-based services. The purpose of this study was to compare occupational therapy students’ perceptions and expectations prior to placements with their perceptions and satisfaction levels upon completion of placements, and to compare clinical placement models (role-established and role-emerging).

**Methods:**

The study included 155 undergraduate occupational therapy students, who completed questionnaires prior to and upon completion of their placements. The questionnaire included items that were divided into categories of placement setting and supervision, personal skills, professional skills, and community. Paired t-tests and two-way repeated measures analyses of variance (ANOVA) were used in order to examine the change in scores over time, and a-parametric tests were used in order to compare the two models.

**Results:**

For all students a significant decrease in scores was found from pre- to post-placement regarding setting and supervision (t[df] = 3.96[154], *p* < .001), and a significant increase in scores was found from pre- to post-placement regarding personal and professional skills (t[df] = 7.82[154], *p* < .001; t[df] = 14.24[154], *p* < .001, respectively). Comparison between placement models indicated nonsignificant differences regarding personal and professional skills. However, role-established post-scores were significantly higher than role-emerging scores regarding setting and supervision, but significantly lower regarding the contribution of services to the community.

**Conclusions:**

Practice placements promote a student’s personal and professional skills. Students were less satisfied with the setting and supervision in comparison to their pre-placement expectations. Both models may contribute to students’ professional development. Role-established model had an advantage in students’ satisfaction with settings and supervision, while role-emerging models had an advantage in students’ perceptions regarding contribution to the community. Role-emerging model may contribute to developing and expanding areas of practice in the community.

**Electronic supplementary material:**

The online version of this article (10.1186/s12909-019-1762-0) contains supplementary material, which is available to authorized users.

## Background

Practice placement education is a fundamental component in the development of students’ personal and professional identity [1, 2]. Practice placement education provides students with the opportunity to apply, validate, and integrate what is learned in the classroom, and to develop clinical reasoning, competence, and self-confidence. It enables opportunities for practice and application of theoretical knowledge with clients across the lifespan in a variety of settings [3–5]. Practice placements build and develop students’ professional and personal skills [4, 6, 7]. Professional development occurs during practice in context and includes development of professional skills (such as application of models and theories, clinical reasoning, and evidence-based practice) and personal skills (such as interpersonal abilities, communication methods, learning and adaptation styles, reflective practice, and use of feedback) [1, 2]. It was found that increasing the students’ personal search for meaning and motivation, as well as reducing their fear of failure, can improve their professional and academic performance [8]. Practice placement experiences lead the student to experience significant changes in the ways they understand their identity and behavior. Students gain resourcefulness, a sense of social duty, self-directed learning, and self-awareness [9], which help them to understand the therapeutic use of self and the ability to foster healthy interactions with clients [9–11]. Mattila and Dolhi (2016) [11] found that practice placement experiences assist students to generalize learned skills and activities, and to transfer those skills to varying populations or clinical settings.

The literature suggests that there needs to be extensive research on practice placement education experiences [3, 11, 12], due to a greater demand for placements, a shift in settings [13], and an awareness that practice placements can be a stressful experience [14]. Current research support that practice placements influence students’ knowledge, professionalism, and self-efficacy outcomes [15, 16]. However, there is limited research focused on students’ expectations, degree of satisfaction, and perceptions; and even fewer studies exist on the perceived benefits of practice placements prior to and post placement [4, 12]. Expectations are defined as a person’s beliefs that a certain behavior or outcome will occur as a result of a specific event; satisfaction is defined as an emotional response to the gap between expectations or desires and the perception of what one actually received or gained [17]. Chiang et al. (2012) [18] found that students’ perceptions of occupational therapy were a significant predictor for their degree of satisfaction with placements.

Within the practice placements, a variety of clinical supervision models exists between clinical supervisors and students [12, 19], which differ according to the practice placements models. Studies have shown that different practice placement models are necessary to ensure that students have the skills and confidence to engage in emerging opportunities, benefit from exposure to learning experiences, and develop critical understanding of their profession [3, 5, 12, 13, 20–22].

Clinical placement models can be classified as either role-established, a more traditional placement; or role-emerging, a nontraditional model. The role-established placement model occurs in various recognized and approved clinical settings where occupational therapists work, such as medical and educational institutions, as well as community services [7, 20, 23]. In the role-emerging placement model, occupational therapy is not routinely provided, but the potential exists [24]. Students develop community-based occupational therapy programs (e.g., community centers for senior citizens or people with a mental illness, homeless shelters, and kindergartens for at-risk children); the supervision is provided daily by an on-site professional (not an occupational therapist) and weekly by an off-site occupational therapist [3, 7, 12, 21, 22, 25, 26]. The use of the role-emerging model is in accordance with the belief that skills and training can and should be used to develop and expand established, emerging, and re-emerging areas of practice [27]. Role-emerging placements, which allow students to explore, establish, and expand roles in the community, have been found to enhance students’ independence, clinical reasoning, problem-solving, and communication skills, as well as broaden their autonomy, self-directed learning, and ability to advocate [5, 20, 28].

Studies among students in role-emerging placements used a small number of students [5, 29] or were related only to one specific area of practice [26]. A number of studies have reported larger numbers of students in more diverse settings [7, 22]; however, in these studies students were surveyed after their placements, offering only a reflective account of their experiences. Only a few studies compared models of practice placement. For example, Gat and Ratzon (2014) [30] compared students’ perceptions of professional and personal skills and reported nonsignificant differences between models. In contrast, Friedland et al. (2001) [22] found that students valued the development of clinical skills in role-established medical placements over those acquired in community placements. Linnane and Warren’s (2017) [31] study highlighted concerns of the role-emerging model relating to the supervision (such as lack of an on-site qualified therapist providing role modelling) and setting (such as communication difficulties), which may not fully grasp the role of occupational therapy and can result in false student expectations.

The purpose of this study was to explore the occupational therapy students’ perceptions, expectations, and satisfaction levels in relation to their practice placements prior to and after completion of placements. Specifically, we aimed to examine: (1) students’ expectations prior to placement, compared with their levels of satisfaction with setting and supervision after completion of placement; and (2) students’ perceptions prior to and after completion of placement regarding their personal and professional skills. Additionally, we compared two models of practice placement (role-established, role-emerging) regarding students’ perceptions and satisfaction levels after completion of the placement, in relation to setting and supervision, personal and professional skills, and contribution of services to the community.

## Method

### Research design

The study was conducted among undergraduate occupational therapy students using a pre-post group design [32], as well as comparisons between groups (models of practice placement). All students who provided written informed consent for their participation were included. The study was approved by the University Ethics Committee.

### Participants and procedure

Participants included 155 undergraduate occupational therapy students from the School of Occupational Therapy of the Hebrew University, spanning two academic years (2015–2017). As part of their educational curriculum, students were required to complete a minimum of 1000 clinical hours guided by the World Federation of Occupational Therapy [33], and complete three practice placements consecutively in the second, third, and fourth years of the program in various practice areas (pediatrics, mental health, physical, and geriatrics). The first practice placement focuses on attaining basic skills in communication, and on the process of evaluation, while the other two practice placements focus more on developing clinical reasoning skills and pragmatic aspects of intervention. During the practice placement period, students are under the supervision of certified occupational therapists who provide ongoing supervision, in coordination with university-affiliated supervisors who follow the students’ personal development continuously throughout all placements. Students participated in one of the two placement models (128 students in role-established practice placements and 27 students in role-emerging practice placements). See Table 1 for descriptive statistics of the participants.

**Table 1:** Descriptive statistics of the participants

	*N* (%)
Gender	
Males	4 (2.6)
Females	151 (97.4)
Practice placement level	
First	64 (41.3)
Second	53 (34.2)
Third	38 (24.5)
Practice placement areas	
Pediatrics	46 (29.7)
Mental health	55 (35.5)
Physical and Geriatrics	54 (34.8)

Note. *N* = 155

### Measure

In order to evaluate students’ perceptions, expectations, and satisfaction levels, we gathered relevant items based on the literature e.g., [30], clinical experience, and reviews from faculty, supervisors, and students. Two versions of the questionnaire were designed for use prior to and upon completion of placements. The preplacement questionnaire included items that were written as outcome measures in order to predict future behaviors (for example: “I expect that the supervisor will provide individualized, direct, specific supervision”) and evaluated students’ perceptions about their personal and professional skills (for example: “I think I have enough theoretical knowledge to succeed in the placement experience”). The post-placement questionnaire included items that evaluated the change in the predicted behaviors during the placement experience (for example: “The supervision I received during the placement experience enhanced my professional knowledge and skills”), as well as students’ perceptions regarding the contribution and importance of occupational therapy services to the community (for example: “I think that my work during the placement contributed to the understanding of occupational therapy services in the community”). For the questionnaires’ categories and items see Additional file 1.

Each version of the questionnaire included 25 items, using a 5-point Likert scale (1 = not at all; 5 = extremely). The items were divided to three distinct categories: (1) placement setting and supervision (10 items); (2) personal skills (9 items); and (3) professional skills (6 items). The post-placement questionnaire also included 5 items related to the additional category of community, which were based on the students’ practice placement experience. Evaluation of the initial psychometric properties of the placement questionnaire included internal consistency, which was found to be high for all questionnaire categories (Cronbach’s α = .845, .767, .831, .754).

### Data analysis

Statistical analyses used the Statistical Package for the Social Sciences (SPSS- Version 24). Descriptive statistics were used and the significance level was set at 0.05. Internal consistency was evaluated using Cronbach’s α for each questionnaire category.

First, regarding all samples, we used a paired t-test in order to examine the change in scores over time (from pre- to post-placement) in the category of placement setting and supervision. Cohen’s d was used to examine the effect size (small = .2 to .4, medium = .4 to .7, and large > .7) [34, 35]. In addition, we used two-way repeated measures analyses of variance (ANOVA) with Bonferroni pairwise comparisons in order to examine the change in scores over time in the categories of personal and professional skills, which were both related to students’ skills. Partial eta squares (*Partial* η^2^) were used to examine overall effect sizes associated with the repeated measures ANOVA (effect size indices for *Partial* η^2^ were: small = .01 to .05, medium = .06 to .13, and large ≥.14) [36]. In order to examine the differences in scores over time in the categories of personal and professional skills, we also used paired t-tests with a Bonferroni correction (significant level for this analysis was *p* < .025 [.05/2]), using Cohen’s d to examine the effect size [34, 35]. Finally, one-way repeated measures ANOVA were also used to examine the differences in scores regarding placement levels, using post-hoc tests with Bonferroni pairwise comparisons.

Second, regarding the two models of practice placement, a comparison was conducted between the post-placement scores of the two models based on nonsignificant (*p* > .05) differences that were found between the pre-scores. Due to unequal sample sizes from the two placement models (role-emerging, role-established), as well as abnormal distribution, we used a-parametric tests (Mann-Whitney tests) in order to compare the post-scores of the two models in each category.

## Results

### Placement setting and supervision

In comparing all students with regard to placement setting and supervision, the results of the paired t-test indicated significant differences with a medium effect size (t[df] = 3.960[154], *p* < .001, Cohen’s d = .44) between the pre- and post-placement scores. The prior-to-placement scores (M = 4.37, SD = .45) were found to be significantly higher than the after-placement scores (M = 4.12, SD = .68). See Table 2.

**Table 2:** Comparison between pre- and post-placement scores of all students

Categories	*N*	Pre	Post	t (154)	Cohen’s d	*p*
		M (SD)	M (SD)			
Settings and supervision	155	4.37 (.45)	4.12 (.68)	3.96	.44	<.001
Personal skills	155	4.06 (.38)	4.34 (.38)	7.825	.737	<.001
Professional skills	155	3.46 (.55)	4.05 (.45)	14.24	1.17	<.001

Note. *M* = mean, *SD* = standard deviation, *t(df)* = Paired sample t-tests, *Cohen’s d* = effect size

### Personal and professional skills

In comparing the pre- and post-placement scores of all students with regard to their personal and professional skills, the results of the repeated measures ANOVA indicated a significant time and category effect, with large effect sizes (F = 177.30[1154], *p* < .001, Partial η^2^ = .54; F = 192.64[1154], *p* < .001, Partial η^2^ = .56, respectively). In addition, the interaction effect between time and category showed a large effect size (F = 56.44[1154], *p* < .001, Partial η^2^ = .27). As can be seen in Table 2, the results of the paired t-tests indicated that in both categories of personal and professional skills, the prior-to-placement scores were significantly lower than the after-placement scores, with a large effect size.

The results also indicated that the personal skills scores in general (M = 4.20, SD = .31) were significantly (*p* < .001) higher than the professional skills scores (M = 3.76, SD = .42), and the change in scores over time (from pre- to post-placement) in the professional skills (from M = 3.46 to M = 4.05; d-score = .59) was higher than the score change in personal skills (from M = 4.06 to M = 4.34; d-score = .28).

### Differences between practice placement levels

The results of the repeated measures ANOVA for each of the three categories indicated significant differences between placement levels only regarding the professional skills, with a large effect size (F = 15.648 [2151], *p* < .001, Partial η^2^ = .172). The results of the post-hoc test indicated that in this category, students’ scores in the first placement level (*n* = 64; pre-score: M = 3.16, SD = .55; post-score: M = 3.92, SD = .42) were significantly lower (*p* < .001) than those of students in the other two placement levels (second level: *n* = 53; pre-score: M = 3.70, SD = .44; post-score: M = 4.11, SD = .43; third level: *n* = 37; pre-score: M = 3.63, SD = .55; post-score: M = 4.19, SD = .48).

### Comparison between the two models of practice placement (role-emerging, role-established)

Items regarding contribution to the community were added to the post-placement questionnaire, due to the fact that these items were relevant only to students upon completion of their practice placement. As such, comparison between the two models of practice placement included four categories (placement settings and supervision, personal skills, professional skills, and community). The results of differences between the pre-scores of the categories were nonsignificant (*p* > .05), and for that reason only the post-scores of the two models of placement were compared. As can be seen in Table 3, the results of the Mann-Whitney tests indicated that in the category of *placement setting and supervision*, the role-established placement scores were significantly (*p* < .001) higher than the role-emerging placement scores. However, in the categories of *personal and professional skills*, the results indicated nonsignificant differences (*p* > .05) between the role-established placement scores and the role-emerging placement scores. In the category of contribution to the *community*, the results indicated that the role-established placement scores were significantly (*p* < .05) lower than the role-emerging placement scores.

**Table 3:** Comparison between the two models of practice placement: students’ post-placement scores

Category	Role-established placement			Role-emerging placement			Z	*p*
	*N*	M	SD	*N*	M	SD		
Placement settings and supervision	128	4.23	.61	27	3.60	.75	−4.10	<.001
Personal skills	128	4.32	.39	27	4.41	.37	−1.10	NS
Professional skills	128	4.05	.47	27	4.05	.33	−.29	NS
Community	128	3.81	.56	27	4.08	.37	−2.35	.019

Note. *M* = mean, *SD* = standard deviation, *Z* = Mann-Whitney tests, *NS* = Nonsignificant (*p* > .05)

## Discussion

This study focused on the perceptions, expectations, and satisfaction levels of undergraduate occupational therapy students prior to and upon completion of practice placements. The first two aims of this study were to compare all students’ perceptions and expectations prior to placements with their perceptions and satisfaction levels after placements, in relation to placement setting and supervision, and personal and professional skills.

### Placement setting and supervision

The results showed a significant decrease in scores from pre- to post-placement, with a medium effect size. These results indicated that all students expressed lower satisfaction with the settings and supervision in comparison to their expectations prior to placements. The gap between expectations and satisfaction levels supports another study in which expectations were high and inconsistent with their actual sense of satisfaction [18]. Although students’ expectations prior to placements were high, and while there was a decrease in the overall scores post-placement, satisfaction levels remained high.

Our finding suggests that prior to placements, students expect high levels of support from supervisors and settings. These expectations include a comprehensive orientation, matched expectations, and individualized supervision that supports academic learning. These finding are supported by the literature [4, 7, 16]. Rodger’s (2011, 2014) [4, 16] work found that the factors crucial for students’ satisfaction and for quality practice placements were opportunities for learning experiences, modeling clear communication, understanding of learning styles, mutual respect, and constructive feedback. Since clinical education studies also expect students to be able to adapt to the existing setting and learn from the supervision provided to them [37], this should be emphasized when preparing students for practice placement.

### Personal and professional skills

The results indicated a significant increase in scores from pre- to post-placement for both personal and professional skills, with large effect sizes. These results support the contribution of practice placements to students’ professional behavior and personal practical skills [2, 11]. The results also indicated that the personal skills scores were significantly higher than the professional skills scores. Significance increased in personal skills, most likely the result of an ongoing personal skill development process that is supported by university-affiliated supervisors. This support of students’ personal skills starts in the first year and continues throughout all practice placement periods. In conjunction with the practice placement supervisor, this university-based support is geared to enhance personal development. With regard to professional skills, the proficiency of the skills varied, due to the context of each practice placement, different models and theories that were used in each setting, and level of clinical reasoning and evidence-based practice.

The results also indicated a significant interaction effect between time and category, with a large effect size. The increase in scores over time in students’ professional skills was higher than that of their personal skills. These results support the assumption that the practice placement experience in and of itself influences students’ perceptions of their skills, particularly their professional skills [1, 12, 26, 38]. Additionally, the results indicated significantly lower scores regarding professional skills of students in the first placement level, compared with other placements levels. These results are reasonable, due to the fact that an improvement in students’ professional skills is expected over the duration through experience in practice placements, and the fact that the first practice placement focuses more on the evaluation process, and less on clinical reasoning and intervention.

### Models of practice placements

The third aim of this study was to compare the two models of practice placement (role-established, role-emerging) regarding students’ perceptions and satisfaction levels after completion of placement. The results indicated that the role-established placement scores after placement were significantly higher than the role-emerging placement scores after placement, with regard to *practice setting and supervision*. It seems that students in a traditional role-established placement, where occupational therapists already work, were more satisfied with the setting and the supervision, compared to students in a role-emerging placement. These results can be explained by the fact that in the role-emerging placement model, occupational therapy is only provided weekly by an off-site occupational therapist, based on a consultative model [3]. Therefore, students do not receive immediate feedback from their supervisors. These results support some concerns regarding the lack of on-site supervision in the setting of role-emerging practice placements [31].

When comparing role-established placement to role-emerging placement, the results indicated nonsignificant differences between students’ perceptions and satisfaction levels after placement, regarding *personal and professional skills.* These results support findings from previous studies also showing nonsignificant differences between role-established and role-emerging models [30]. However, these results do not support findings indicating the advantages of role-established placements over role-emerging community placements in developing clinical skills of students [22]. The results of this study indicated that both practice placement models contribute to the development of personal and professional skills. They therefore support the belief that the role-emerging model can also be used as an appropriate model [39] and may even contribute to developing and expanding areas of practice in a community [27], including private practice [40].

In comparing students’ perceptions upon completion of placements, regarding the contribution and importance of occupational therapy services to the *community,* the role-established placement scores were significantly lower than the role-emerging placement scores. These results can be explained by the fact that the focus in a role-emerging placement is development of community-based occupational therapy programs [3]. Therefore, students who participate in a role-emerging placement may have more opportunity to explore, establish, and expand roles in the community [28, 38, 41].

#### Implications for occupational therapy education

It is recommended to consider developing role-emerging placements, and to encourage students to be more involved in developing services in the community by increasing their awareness and knowledge regarding the contribution of occupational therapy in developing community partnerships. Educators and placement coordinators should choose appropriate placements and clinical supervision models, and also may consider a combination of different practice placement models. One example is an interagency model, which is a combination of the apprenticeship model (one student to one supervisor) and the consultative model (students divide their time between an established placement and an emerging placement in the community) [12, 19].

Students should be given the skills to work autonomously, thus encouraging independence and more self-directed learning. Participation in a role-emerging placement can be a positive learning experience for students who express an interest in working with populations in the community. Better prior preparation of students, placement settings and supervisors is essential, especially in role-emerging placement. Educators and university-affiliated supervisors need to ensure that students have realistic expectations from supervisors and settings. Finally, encouraging better communication between university-affiliated professionals and placement supervisors is vital. Settings that incorporate occupational therapy students must understand that role-emerging practice placements require staff with the ability to devote additional time and resources to supervising students.

#### Limitations and future research

In interpreting this study’s findings, several limitations should be taken into account. First, there was an unequal representation of students in role-emerging placements compared to role-established placements. This can be explained by the fact that greater resources need to be allocated in order to expand the numbers of students who participate in role-emerging placements, compared to role-established placements. Additionally, different variables (such as types of placement settings, clinical supervision models, and supervisors’ experience) may also influence students’ perceptions and satisfaction levels; these variables need to be further investigated, using a large number of students who participated in both practice placement models. We examined only the initial psychometric properties of the questionnaire; additional psychometric properties should be investigated in future studies, as well as using qualitative data. Additionally, due to the importance of developing practice placements in the community, future research should also include perceptions and satisfaction levels of supervisors and community partnerships, regarding the contribution of occupational therapy students to the community, as well as the cost-efficiency in developing new services.

## Conclusions

This is the first study examining students’ perceptions and expectations prior to placement, compared with their perceptions and satisfaction levels after completion of placements, as well as comparing different models of practice placements, and their contribution to the community. The results of this study support the belief that clinical practice placements contribute to the personal and professional skills of occupational therapy students. The results also support the students’ needs for professional supervision and for suitable practice placement settings that provide support and preparation prior to their arrival, as well as encouraging their independence and self-directed learning.

The results of this study reinforce the knowledge that continued supervision for students from their first year through all practice placement periods enhances students’ personal development. Student levels of satisfaction are directly related to learning experiences in practice placements. Thus, structured supervision and better preparation of students, especially in role-emerging placements, will ensure that their expectations are realistically met. Finally, role-emerging practice placements contribute to the developing of students’ skills, as well as their perception regarding engagement in the community. It is necessary to choose appropriate placements, as well as to expand placements in the community, using a variety of clinical supervision models. Future studies are needed, using a large number of students who participate in role-emerging practice placements, as well as investigating additional psychometric properties of the questionnaires.

## Additional file

Additional file 1: Questionnaire categories and items. Prior to practice placement, students indicated their perceived perceptions and expectations regarding each of the 25 items, using a 5-point Likert scale (1 = not at all; 5 = extremely). Upon completion of their practice placement, students also indicated their level of satisfaction regarding all items, including items related to the category of community. Notes: 1 = Not at all, 2 = Slightly, 3 = Moderately, 4 = Very, 5 = Extremely; *Filled in upon completion of practice placement. (DOCX 18 kb)

## Data Availability

The data used to support the findings of this study are available to the authors, but cannot be released due to ethical guidelines.
