# Supplementary material for: “Perceptions, expectations and satisfaction levels of occupational therapy students prior to and after practice placement and comparison of practice placement models”
Source: BMC Med Educ. 2019 Aug 29;19:324. doi: 10.1186/s12909-019-1762-0 (PMC6716819; doi:10.1186/s12909-019-1762-0)
Supplement: Supplementary file 1 — Questionnaire categories and items. Prior to practice placement, students indicated their perceived perceptions and expectations regarding each of the 25 items, using a 5-point Likert scale (1 = not at all; 5 = extremely). Upon completion of their practice placement, students also indicated their level of satisfaction regarding all items, including items related to the category of community. Notes: 1 = Not at all, 2 = Slightly, 3 = Moderately, 4 = Very, 5 = Extremely; *Filled in upon completion of practice placement. (DOCX 18 kb) [file 12909_2019_1762_MOESM1_ESM.docx]

**Additional file**

**Questionnaire categories and items**

Prior to practice placement, students indicated their perceived perceptions and expectations regarding each of the 25 items, using a 5-point Likert scale (1 = not at all; 5 = extremely). Upon completion of their practice placement, students also indicated their level of satisfaction regarding all items, including items related to the category of community.

| **Category** | **Item** | **1** | **2** | **3** | **4** | **5** |
| --- | --- | --- | --- | --- | --- | --- |
| Practice placement and supervision  (10 items) | 1. Orientation process in the Setting |  |  |  |  |  |
|  | 1. Matched expectations with supervisor. |  |  |  |  |  |
|  | 1. Matched expectations of practice placement. |  |  |  |  |  |
|  | 1. Adequate amount and type of supervision. |  |  |  |  |  |
|  | 1. Information about setting policies, procedures. |  |  |  |  |  |
|  | 1. Supervisor provides individualized, direct, specific supervision. |  |  |  |  |  |
|  | 1. Setting supports academic learning. |  |  |  |  |  |
|  | 1. Supervisor competency in professional knowledge and skills. |  |  |  |  |  |
|  | 1. Interpersonal and professional support from staff members. |  |  |  |  |  |
|  | 1. Staff preparation. |  |  |  |  |  |
| Personal abilities  (9 items) | 1. Well integrated into practice placement. |  |  |  |  |  |
|  | 1. Collaborative relationship with staff and population. |  |  |  |  |  |
|  | 1. Collaborative relationship with the supervisor. |  |  |  |  |  |
|  | 1. Ability to receive constructive feedback. |  |  |  |  |  |
|  | 1. Opportunities to work creatively. |  |  |  |  |  |
|  | 1. Opportunities to work independently. |  |  |  |  |  |
|  | 1. Opportunities to initiate new innovative ideas. |  |  |  |  |  |
|  | 1. Ability to solve problems through clinical reasoning. |  |  |  |  |  |
|  | 1. Ability to ask questions. |  |  |  |  |  |
| Professional abilities  (6 items) | 1. Competency in theoretical knowledge. |  |  |  |  |  |
|  | 1. Competency in evaluation process. |  |  |  |  |  |
|  | 1. Competency in intervention process. |  |  |  |  |  |
|  | 1. Collaborates with others (colleagues, family/support system, and other staff). |  |  |  |  |  |
|  | 1. Professional contribution to the practice placement. |  |  |  |  |  |
|  | 1. Articulates and implements occupational therapy role. |  |  |  |  |  |
| Community*  (5 items) | 1. Contribution of knowledge and experience to the community. |  |  |  |  |  |
|  | 1. Continuity of the services that were developed during the practice placement. |  |  |  |  |  |
|  | 1. Enhance the understanding of occupational therapy’s role of contribution to the community. |  |  |  |  |  |
|  | 1. Commitment to contribute to the community. |  |  |  |  |  |
|  | 1. Partnering in developing community-based occupational therapy services. |  |  |  |  |  |

Notes: 1= Not at all, 2 = Slightly, 3 = Moderately, 4 = Very, 5 = Extremely; *Filled in upon completion of practice placement.
